# Parental perspectives on the quality of life of children with Down syndrome

**DOI:** 10.3389/fpsyt.2022.957876

**Published:** 2022-08-12

**Authors:** Elisa Fucà, Paolo Galassi, Floriana Costanzo, Stefano Vicari

**Affiliations:** ^1^Child and Adolescent Neuropsychiatry Unit, Department of Neuroscience, Bambino Gesù Children's Hospital, IRCCS, Rome, Italy; ^2^Department of Life Science and Public Health, Catholic University of the Sacred Heart, Rome, Italy

**Keywords:** trisomy 21, analogical reasoning, challenging behaviors, ritualistic behavior, autistic symptoms

## Abstract

Down Syndrome (DS) is the most common chromosome abnormality and the most frequent cause of developmental delay/intellectual disabilities in children. Although the investigation of the quality of life (QoL) is crucial in children with DS, relatively poor attention has been paid to this topic. The current study aimed to evaluate parent-reported QoL in a group of children with DS and identify children's individual and clinical features associated with different levels of QoL. We included in the study 73 children with DS (5–12 years) and investigated the parent-reported levels of QoL by means of the Pediatric Quality of Life Inventory. Cognitive level and the presence of behavioral difficulties were also evaluated. The overall parent-reported QoL of children with DS was high; emotional functioning was the domain with the highest level of QoL. Moreover, parents perceived low levels of QoL in children who exhibited low IQ, worse analogical reasoning, worse adaptive skills, more frequent challenging behaviors, more ritualistic/sameness behavior and more autistic symptoms. No differences emerged for family variables, namely parental education and employment, between the two groups with high and low QoL, as perceived by parents. The understanding of cognitive and behavioral factors - such as analogical reasoning, socio-communication abilities and challenging behaviors - related with different degrees of QoL in children with DS is crucial for the development of effective strategies to promote the improvement of the QoL.

## Introduction

According to the World Health Organization, *quality of life* (QoL) can be defined as “individuals' perceptions of their position in life in the context of the culture and value systems in which they live in relation to goals, expectations, standards, and concerns” (1). The assessment of QoL includes the investigation of the individual's or caregiver's subjective evaluation of well-being across different domains, such as physical, emotional and social well-being. This model allows to measure subjective estimates of QoL across individuals at all developmental stages and across individuals with and without developmental disabilities. In particular, evaluation of QoL among children and adolescents is critical in detecting subsets with poor health status and in developing effective strategies to increase health of the younger population (2). The importance of a proper assessment of QoL in pediatric age becomes even clearer with reference to clinical groups. In these cases, evaluation of QoL in clinical practice can help optimizing communication between clinicians and the child and his/her caregivers as well as recognizing physical or mental health problems from the patient and caregiver perspectives and areas of potential amelioration (3).

The assessment of QoL is well-suited to conditions that have a multifaceted impact, such genetic syndromes and neurodevelopmental disorders, including intellectual disability (ID).

Children with these conditions manifest several difficulties in communication, motor and social functioning, with important consequences on the possibility to independently manage daily life activities. Consistently, children with neurodevelopmental disorders have higher health care service utilization than typically developing children (4, 5) and are more vulnerable to mental health problems (6). Given such a multidimensional impact of neurodevelopmental disorders, increasing attention has been paid to the investigation of QoL in children with neurodevelopmental disorders and genetic syndromes. In particular, children with ID display poorer QoL than their typically developing peers (7); similarly, parents of children with neurogenetic syndromes, such as Prader Willi syndrome, tend to perceive lower levels of QoL in their children (8). Research identified a number of factors associated with poorer QoL, such as older age, worse adaptive skills, the presence of challenging behaviors, and more complex needs such as autism spectrum disorder and medical comorbidities (9–19). For instance, Beadle-Brow and collaborators (12) reported life satisfaction, as perceived by proxy respondents, was related to independent functioning in childhood in a cohort of people with severe ID and/or autism; moreover, the same study provided support for a crucial role of challenging behaviors in the proxy respondents' perception of QoL. These results were confirmed and extended by a subsequent study including a group of 246 children with severe developmental disabilities that reported that higher QoL was related to younger age, higher adaptive skills, lower maladaptive behavior (7). The central role of adaptive behavior was further supported by another study on adults with intellectual and developmental disabilities; the authors found not only adaptive behavior was significant and relevant from both third-party and participants' perspective, but also problem behaviors had a modest negative impact on the QoL (17). Similar results were reported also from research on specific neurogenetic syndromes, such as Fragile X. Coffman and colleagues (19), indeed, found parent-reported QoL significantly correlated with adaptive functioning, social impairment, and aberrant behaviors; in particular, greater parent-reported QoL was associated with reduced aberrant behaviors. Findings on challenging behaviors are highly relevant with reference to QoL, given that individuals with ID are three times as likely to exhibit aggressive behaviors toward others, self-injury, or behavior destructive to property (20). To address such complex needs in this population, targeted interventions are designed to support children and their families globally.

Among children with developmental disabilities, those with Down Syndrome (DS) exhibit specific features. With a worldwide incidence rate of 1:1,000–1,100 in newborns (21), DS is the most common chromosome abnormality and the most frequent cause of developmental delay/ID in children (22). DS is caused by the presence of a supernumerary chromosome 21, resulting in a constellation of clinical features (23). This includes ID often associated with multiple health comorbidities such as cardiac defects, delayed growth, hematology and thyroid abnormalities, autoimmune diseases, and obstructive sleep apnea (23), as well as with behavioral problems (24, 25). Such a complex condition contributes to the demand for additional medical care in comparison with other forms of ID. Considering the important consequences deriving from cognitive, medical and behavioral correlates of DS on individual's functioning, it is crucial to measure the QoL in such population, as well as to identify factors that can influence it.

Despite the investigation of the QoL is crucial in children with DS, relatively poor attention has been devoted to this topic (23). However, existing research indicates that QoL of children with DS is lower than typically developing children (26–28) and, more specifically, variations in the levels of QoL across different domains emerge. For instance, children with DS exhibit low levels of physical well-being but high levels of emotional well-being (28, 29). Concerning the factors associated, sex seems to be unrelated to QoL in DS (30, 31), whereas findings on age are inconsistent. Indeed, some studies indicated a worsening of QoL with increasing age (29, 31), others reported higher QoL in young adults in comparison with adolescents (30), and others failed to detect age-related differences (32). Moreover, behavioral problems and medical comorbidity have been associated with poorer QoL in adolescents with DS (30). Finally, family resources, such as family income, have been also related to QoL in children with DS (33).

In spite of the increased awareness of the importance of evaluating QoL in children with DS, research investigating the levels of QoL and associated factors is scarce. In addition, inconsistent findings about the correlates of QoL in children with DS still persist. Finally, few evidence derives from the evaluation of objectively measured correlates of QoL in children with DS, such as IQ. Indeed, most of the previous studies mainly rely on parental reports on child's QoL and used a single instrument for behavioral evaluation excluding a detailed characterization of specific behavioral aspects - including challenging behaviors and autistic symptoms.

Specific aims of the present study were: (i) evaluating parent-reported QoL in a group of children with DS; (ii) identifying child's individual and clinical features associated with different levels of children's QoL as perceived by parents. Based on previous research on neurodevelopmental disorders, we hypothesized that lower QoL levels in the physical domain than in the emotional and social domains would be observed. We also hypothesized that the children perceived as with higher QoL would have higher cognitive abilities, better adaptive skills, less autistic symptoms, less behavior problems and less repetitive behaviors.

## Materials and methods

### Participants

Seventy-three children with DS (46 boys, 27 girls) ranging in age from 5 to 12.11 years of age (mean 8.97 ± 2.24 years) were included in the study. The mean IQ was 57.42 ± 7.08. Selection criteria included, besides the diagnosis of DS based on the analysis of the karyotype, the age ranging between 7 and 12 years. Exclusion criteria were as follows: age <5 and >12 years; language barrier hampering questionnaire compilation by parents. All participants underwent a child psychiatric and neuropsychological examination conducted by experienced developmental neuropsychiatrists and neuropsychologists.

### Procedure

This is a cross-sectional study; data were retrospectively collected from a file review of patients with DS referred for a clinical evaluation at the Child and Adolescent Neuropsychiatry Unit of the Bambino Gesù Children's Hospital in Rome between December 2021 and April 2022. We investigated the levels of parent-reported QoL in children with DS; moreover, the differences between children with high levels of QoL and children with low QoL were analyzed. Child's characteristics included sex, age, non-verbal IQ, adaptive level, behavioral problems and autistic symptoms. The clinical evaluation of children and adolescents with DS consisted in a neuropsychiatric, neuropsychological and psychopathological/behavioral assessment performed by a team made of a child neuropsychiatrist and clinical psychologists and neuropsychologists with clinical expertise on DS. The clinical evaluation also included the administration of parent-report questionnaires, which were filled out by the parents while the children underwent neuropsychological or behavioral evaluation. All parents received precise instructions regarding filling out the questionnaires.

Due to the retrospective design, data were collected from the hospital records and clinic charts and the de-identified data were analyzed. All parents signed a written informed consent for data use for research purposes and a privacy statement that ensures that data will be kept confidential. For the current project, all subjects meeting specified criteria as described above were selected from a database. The study was conducted according to the guidelines of the Declaration of Helsinki.

### Measures

#### Quality of life

Pediatric Quality of Life Inventor (PedsQL™) 4.0 is the standard generic core scale of QoL to measure the physical and psychosocial health of either healthy or ill children (34). The questionnaire was administered to parents. PedsQL has been previously used to assess parent-reported QoL also in children with DS (27, 28) and other forms of developmental disabilities (35). The instrument is age-specific: for this study, we used the 5–7 years and the 8–12 years versions. The PedsQL includes 23 questions with a five-point rating scale (range of 0 to 4 points based on the agreement in each statement). Questions cover the following areas: physical health and psychosocial health, including emotional, social, and cognitive/school functioning. The scores were transformed into full scores of a maximum of 100 points in each area: 100 points meant good QoL, while 0 points meant QoL with very frequent problems. The reliability and validity of PedsQL generic core scales have been well established in healthy and sick populations (36–39). There are no cutoff points to distinguish high QoL from low QoL. However, different studies have tried to distinguish between good and poor QoL. Cut offs for the current study were chosen to be based on the study by Varni and Limbers (34), who identified child self-reported total score of 69.1 and parent proxy reported total scores of 65.4 as “meaningful cut off points for impaired QoL,” at one standard deviation below the average PedsQL score for healthy children. We considered the mean between these two reported cut-offs (i.e., 67.2, approximated at 67) to identify high levels of QoL.

#### Cognitive abilities

Cognitive development was tested by the Leiter-3 (40),which provides a nonverbal measure of intelligence and assesses the ability to reason by analogy, matching and perceptual reasoning, irrespective of language and formal schooling for individuals ages 3–70.

#### Adaptive functioning

To assess the presence of impairments in adaptive behaviors necessary for socialization, communication, and daily functioning, we used the Adaptive Behavior Assessment System II (ABAS-II) (41), a parent/primary caregiver questionnaire. ABAS-II consists of eleven skill areas organized into three general domains: conceptual, practical, and social. The composite and domain scores are standard scores with a norm referenced mean of 100 and standard deviation of 15. ABAS-II was standardized and validated for Italian population, showing high internal consistency, good levels of reliability and convergent and clinical validity.

#### Behavioral problems and autistic symptoms

Aberrant Behavior Checklist (ABC). The ABC (42) is a caregiver rating scale used to assess the severity of core symptoms and comorbid emotional and behavioral problems for several neurodevelopmental and genetic disorders, including DS (43, 44). It consists of 58 questions and five subscales: Irritability/Agitation/Crying; Lethargy/Social Withdrawal; Stereotypic Behavior; Hyperactivity/Non-compliance; Inappropriate Speech. Several studies by the authors (42, 45) and by independent researchers (46, 47) have supported the reliability and validity of the ABC. Moreover, the instrument was used by its authors who determined a good criterion validity in a population of individuals with DS (48).

Social Communication Questionnaire (SCQ) (49). SCQ is a 40-item parent-report questionnaire investigating three major aspects of autism spectrum disorder: communication, social interaction, and repetitive behaviors. The questionnaire exists in two forms: lifetime and current. The “lifetime” form evaluates the patient's developmental history as well as current behaviors, whereas the “current” form assesses the child's behavior during the past 3-month period only. In the present study, the “lifetime” form was used. Item level validity is good (50, 51); sensitivity and specificity in school-aged samples are relatively high (0.86 and 0.78, respectively) (52); specificity and sensitivity >0.80, together with good convergent and discriminant validity, have been reported in a large sample of children with DS (53).

Repetitive Behavior Scale-Revised (RBS-R). The RBS-R (54) is a clinical rating scale including 43-items in a caregiver-completed questionnaire. Items are grouped into six subscales: (1) Stereotyped Behavior; (2) Self-Injurious Behavior; (3) Compulsive Behavior; (4) Ritualistic Behavior; (5) Sameness Behavior; and (6) Restricted Interests Behaviors. Examination of the psychometric properties of the RBS indicates that item-level inter-rater and test-retest reliability and validity are good (55). Previous research found five RBS-R factors (compiling Ritualistic and Sameness domains together) (56). Since the use of the Italian version of the instrument has not been consistently applied in preschoolers (57), we administered RBS to parents of children from 6–12 years of age.

#### Family variables

Parental education and parental employment were, respectively collected for fathers and mothers and considered as family resources and income indicators, possibly linked to the perceived children's QoL. Parental education was measured as the number of years of education attained. Parental employment was classified as “Not employed,” “Lower supervisory, technical, (semi) routine, others,” “Intermediate, small employers, own accountants,” and “Managerial/professional.”

### Statistical analyses

Descriptive statistics were used to analyze demographic and clinical characteristics of the whole sample. To surmise the differences of children's features by QoL groups, *t* test and repeated measures analysis of variance (ANOVA) were used; Chi-squared test was used to determine the non-parametric variables. Partial eta squared ($\eta_{p}^{2}$) was used to measure effect size. *Post hoc* analyses were performed using Tukey HSD test. A *p*-value ≤ 0.05 was considered as statistically significant.

## Results

### Quality of life

PedsQL scores are reported in Table 1. Significant differences between domains emerged only for the emotional functioning, which was significantly higher than the other domains (all *p* < 0.001).

**Table 1:** Mean score (standard deviation) of PedsQL, ABAS II, SCQ, ABC, and RBS in the total sample and in the two QoL groups.

**PedsQL**						
	**Total score**	**Physical health**	**Emotional functioning**	**Social functioning**	**School functioning**	**Psychosocial health**
**Total sample**	69.01	67.6	77.53	67.57	63.78	67.44
	(16.45)	(20.41)	(16.54)	(22.19)	(19.26)	(19.41)
**Low QoL group**	52.35	51.26	65.14	47.93	49.71	54.56
	(7.01)	(15.56)	(13.36)	(15.01)	(15.1)	(8.86)
**High QoL group**	81.31	79.68	86.67	82.07	74.17	76.95
	(8.61)	(14.26)	(12.18)	(13.81)	(14.98)	(19.66)
**ABAS II**						**SCQ**
	**Global adaptive composite**		**Conceptual composite**	**Social composite**	**Practical composite**	**Total score**
**Total sample**	50.86		55.68	63.67	53.32	10.22
	(13.15)		(8.96)	(14.02)	(13.91)	(6.25)
**Low QoL group**	46.06		53.29	59.42	48.42	13.35
	(9)		(6.73)	(12)	(10.12)	(6.55)
**High QoL group**	54.4		57.49	66.88	57.02	7.9
	(14.63)		(10.04)	(14.7)	(15.29)	(4.93)
**ABC**						
	**Total score**	**Irritability/Agitation/crying**	**Lethargy/social withdrawal**	**Stereotypic behavior**	**Hyperactivity/non-compliance**	**Inappropriate speech**
**Total sample**	26.28	6.01	3.88	3.57	10.35	2.76
	(24.86)	(6.94)	(5.15)	(4.53)	(9.68)	(5.76)
**Low QoL group**	42.72	10.31	6.34	5.65	16.41	4.72
	(26.31)	(7.92)	(5.88)	(5.03)	(10.39)	(8.11)
**High QoL group**	14.05	2.82	2.05	2.02	5.84	1.31
	(14.85)	(3.78)	(3.64)	(3.43)	(6.06)	(2.19)
**RBS**						
	**Total score**	**Stereotyped behavior**	**Self-injurious behavior**	**Compulsive behavior**	**Restricted interests behaviors**	**Ritualistic/ sameness behavior**
**Total sample**	20.18	3.54	1.32	3.06	2.26	9.24
	(20.02)	(4)	(2.77)	(3.86)	(2.56)	(10.11)
**Low QoL group**	27.03	5.10	1.86	3.76	3.21	13.38
	(21.96)	(4.31)	(2.62)	(4.13)	(2.84)	(11.69)
**High QoL group**	14.67	2.28	0.89	3.62	1.5	7.42
	(16.64)	(3.31)	(2.89)	(0.60)	(2.05)	(7.85)

The participants included in the study were then split into two groups: children with high and low QoL (≤66 PedsQL total score; *N* = 31; Low QoL group) and children with high QoL (≥67 PedsQL total score; *N* = 42; High QoL group). The two groups did not differ for age (*p* = 0.072) nor sex distribution (*p* = 0.793). We have also investigated the presence of differences in family variables potentially influencing parental perception of children's QoL, namely parental education (in years) and family income. We did not detect differences between Low QoL and High QoL groups in both maternal and paternal education (i.e., years of schooling; *p* = 0.257 and *p* = 0.372, respectively). Similarly, differences between groups for parental occupation did not emerge for mothers nor fathers (*p* = 0.252 and *p* = 0.985, respectively). Mean scores and standard deviations of the questionnaires are reported in Table 1.

### Differences in cognitive and adaptive abilities

Children in the High QoL group exhibited significant higher IQ than children in the Low QoL group (M = 59.36, SD = 6.72 and M = 54.81, SD = 6.8, respectively). ANOVA analysis with Group (Low QoL and High QoL) as between factor and the Leiter-3 subtest scores as within factor showed significant differences among the groups, F_(3,204)_ = 3.60, *p* = 0.014, $\eta_{p}^{2}$ = 0.05. *Post-hoc* analyses (Tukey HSD test) revealed that the groups differed only for the Classification and Analogies subtest (M = 2.4, SD = 2.1 for the High QoL group and M = 0.83, SD = 1.2 for the Low QoL group; *p* = 0.001). Moreover, children in the High QoL group displayed higher General Adaptive Composite scores at the ABAS II than children in the Low QoL group (M = 54.4, SD = 14.63 and M = 46.1, SD = 8.1, respectively; *p* = 0.006).

### Differences in behavioral problems and autistic symptoms

ANOVA analysis with Group (Low QoL and High QoL) as between factor and the ABC subscales as within factor showed significant differences among the groups, F_(4,264)_ = 6.938, *p* < 0.001, $\eta_{p}^{2}$ = 0.09. *Post-hoc* analyses (Tukey HSD test) revealed that children in the Low QoL group exhibited significantly higher scores at the Irritability/Agitation/Crying subscale than children in the High QoL group (M = 10.31, SD = 7.92 and M = 2.82, SD = 3.78, respectively; *p* < 0.001) and at the Hyperactivity/Non-compliance subscale (M = 16.41, SD = 10.39 and M = 5.84, SD = 6.06, respectively; *p* < 0.001).

Children in the Low QoL group exhibited significantly higher scores at SCQ than children in the High QoL group, indicating more autistic symptoms in this group (M = 13.35, SD = 6.55 and M = 7.9, SD = 4.93, respectively; *p* < 0.001).

A subsample of 65 parents completed the RBS. ANOVA analysis with Group (Low QoL and High QoL) as between factor and the RBS subscales as within factor showed significant differences among the groups, F_(4,252)_ = 4.191, *p* = 0.003, $\eta_{p}^{2}$ = 0.006. *Post-hoc* analyses (Tukey HSD test) revealed that children in the Low QoL group exhibited significantly higher scores at the Ritualistic and Sameness domain than children in the High QoL group (M = 13.38, SD = 11.69 and M = 7.42, SD = 7.85, respectively; *p* < 0.001).

## Discussion

The aim of the present study was to investigate levels of QoL, as perceived by parents, in a sample of children with DS (ages 5–12 years) and the associated features. Results document how the overall parent-reported QoL of children with DS is high and that emotional functioning is the domain with the highest level of QoL. Moreover, consistently with our hypotheses, children with higher levels of QoL exhibit higher IQ, better adaptive skills, less challenging behaviors, less ritualistic/sameness behavior and less autistic symptoms than children with lower QoL. Moreover, differences in the family variables considered, namely parental education and occupational status, do not emerge in our sample, thus suggesting the identified child's variables result independent from family variables, at least in part.

### Parents report high quality of life in children with DS

Emotional functioning is the domain with the highest levels of QoL reported by parents, whereas school functioning is the domain with the lowest scores. This result is in line with previous research reporting high levels of emotional well-being in children with DS (27, 28). Moreover, lower levels of QoL in physical health domain could be explained in light of the higher likeliness of individuals with DS to develop several medical conditions such as cardiac complications and pulmonary disorders (58).

Differently from previous studies, our results failed to document differences in age between the High QoL group and the Low QoL group. Indeed, worse QoL in adolescents with DS aged 16 to 18 years in comparison with children with DS aged 5 to 12 years was reported by Shields and collaborators (29). Lee and collaborators (31) found how the levels of emotional well-being decreases in the transition from childhood to adolescence, namely, children aged 4–5 years showed higher emotional well-being than adolescents aged 13–21 years. In the present study, the lack of significant differences on age between the High QoL and the Low QoL groups could be due to the more restricted age range of the participants included in the current study in comparison with previous research. On the other hand, findings on sex distribution are consistent with literature reporting no sex differences in QoL of individuals with DS (30, 31).

### High quality of life is perceived in children with good analogical reasoning

Children with higher QoL exhibited significantly higher IQ than children belonging to the Low QoL group; intriguingly, these differences between groups could be ascribed uniquely to higher scores obtained at the Classification and Analogies subtest of the Leiter-3 by the High QoL group. This subtest includes both representational and non-representational reasoning as well as analogical reasoning problems and measures the ability to perceive and use relational similarity between two objects or geometric shapes and to *inductively* generate rules out of partial information (40). Of note, cognitive activities such as categorization, probability judgment, analogical reasoning, scientific inference, and decision-making include inductive reasoning (59). Several abilities, ranging from problem solving to social interaction, contain some forms of inductive and analogical reasoning, using what is known to make inferences about what is unknown and to find similarities (60). In particular, analogical reasoning seems to support the comprehension of the abstract similarities between seemingly different situations, especially social situations (61–64). Therefore, analogical reasoning plays a critical role in social learning, supporting children in handle social interactions (63, 65, 66). In sum, it emerges significant impairment in analogical reasoning leads to lower perceived QoL in children with DS, suggesting potential implications for cognitive intervention not only in the academic and adaptive functioning, but in the overall QoL. However, it is not clear if training analogical reasoning on non-social stimuli may directly extend to social domain. Cognitive training for individuals with DS should then include exercise on analogical reasoning applied to social sphere. Moreover, future research is required to determine whether training reasoning in relation to social and non-social aspects can improve social abilities and QoL in children with DS. It must be acknowledged that the present study identified several other factors possibly contributing to QoL in children with DS; therefore, interventions aiming to improve QoL should adopt a comprehensive approach, focusing on both cognitive and behavioral domains.

Results from cognitive evaluation were mirrored by findings on adaptive functioning, indicating that children with higher QoL had better adaptive skills than children with low QoL. Adaptive behavior includes practical domain (e.g., feeding, personal care, and staying safe), social functioning (e.g., interpersonal skills, understanding and compliance with rules, and resolution of social problems), and conceptual domain (e.g., language and communication, reading and writing, and handling figures) (46). Children with DS could exhibit reduced autonomy due difficulties with language, memory and executive functions (18). These impairments often require parents and caregivers of individuals with DS to provide support for everyday activities. Of note, children who are not autonomous with daily living skills may lose important social and educational opportunities. The association between adaptive behavior and QoL has been reported in other forms of disability, such as congenital visual disorders (67), and in neurodevelopmental disorders, such as ID not associated with DS (7, 17). The present study confirms these findings also in DS population, indicating that child's adaptive skills may play a role in the perception of QoL in parents of children with DS.

### Children with more behavior problems, autistic symptoms, and ritualistic/sameness behaviors are perceived with lower quality of life

As expected, children with perceived lower QoL showed more behavior problems; in particular, significant differences between groups emerged in the Irritability/Agitation/Crying and Hyperactivity/Noncompliance scales of the ABC, with children in the Low QoL group exhibiting higher scores. This is consistent with previous research indicating the impact of behavioral problems on QoL of children with ID (2). Moreover, this finding is in line with literature reporting lower levels of QoL in individuals with attention deficit/hyperactivity disorder, that affects both physical and psychosocial domains (68). Of note, individuals with DS are at risk to exhibit hyperactivity, impulsivity, attentional problems, and non-compliance (69, 70). Given that the presence of behavioral difficulties linked to irritability and hyperactivity may influence the way parents perceive the QoL of their children, it is recommendable that interventions to improve maladaptive behaviors in children with DS should consider also QoL as outcome measure.

As concerns autistic symptoms, children perceived with low QoL exhibited more symptoms as detected by means of the SCQ. This result is consistent with literature reporting lower levels of QoL in children with autism spectrum disorder in comparison with other neurodevelopmental disorders, included DS (71, 72). In addition, previous research reported that children with ID and comorbid autism spectrum disorder exhibit worse QoL than those with only ID; specifically, they display lower physical wellbeing as well as lower scores for interpersonal relationships and social inclusion domains (73). Here, we confirm and extend this result in population with DS. Such finding should be properly took into account when setting up behavioral intervention for children with DS who exhibit autistic symptoms.

As concerns repetitive behaviors, our findings are consistent with previous studies indicating that low QoL is associated with severity of repetitive behavior in children with autism spectrum disorder (74). Of note, in our sample children perceived with low QoL only differed from the High QoL group in the ritualistic/sameness behaviors domain of the RBS-R. This domain captures both the attitude toward the performance of daily living activities in a similar matter and the resistance to change, insisting that things stay the same that can be associated to low cognitive flexibility (56). In the same vein, our findings on cognitive abilities, analogical reasoning and autistic symptoms, related to different levels children's QoL according to parental perspective, suggest that impaired cognitive flexibility could be a crucial factor playing a role for the QoL in children with DS.

The main limitation of the study is that the evaluation of QoL relied on a parent-report instrument and that we missed other sources of information, for example self- or teacher-reports. However, self-report instruments or interviews used to measure the QoL in individuals with ID, included DS, mainly target adult age (75–77). Unfortunately, the use of parent/caregiver-report instruments seems to be the only way to explore the QoL in children with moderate or severe ID. Another important limitation of the study is the lack of a comparison with other groups with different neurodevelopmental disorders. Further research is required to investigate differences and similarities in the determinants of QoL across different forms of developmental disabilities, such as autism spectrum disorder and/or other genetic syndromes. The third limitation of the current study is the cross-sectional design, which prevents drawing conclusions about the nature of the relationship between QoL and the identified variables. Therefore, future studies should aim to identify causal links between cognitive and behavioral factors and QoL levels in children with DS; moreover, longitudinal studies could allow the identification of important predictors of QoL in adolescence and adult age. Moreover, even if the reliability measures of the employed instruments are high according to literature, we did not provide the reliability measures of these instruments in the current study. Finally, given the high proneness of individuals with DS to medical comorbidities, future research on this topic should also consider the influence of physical health problems on QoL levels. It must be noticed that the study was conducted after the onset of the COVID-19 pandemic. Therefore, it cannot be established if, for some individuals, COVID-19 pandemic should have affected QoL values, at least in part. More in-depth research focused on conditions prior to the onset of the pandemic could help us better understand the impact of the pandemic on different domains of QoL in children with DS.

Despite these limitations, to the best of our knowledge this is the first study reporting a characterization of cognitive and behavioral features that play a role in the parental perspective on the QoL of their children with DS. The understanding of factors related with different degrees of QoL in children with DS is important for different reasons. For instance, some authors underlined the importance of measuring the QoL in therapeutic trials for the amelioration of cognitive function in individuals with DS (78). It cannot be excluded indeed that effective treatments, despite inducing higher potential for independence and employment, could make people with DS less satisfied with their lives (78). More importantly, an in-depth understanding of the factors affecting QoL in people with DS could be useful for the identification of strengths and weaknesses of the services provided for this population and, in turn, could help in the developing of effective strategies to promote the improvement of the QoL by addressing unmet needs. Indeed, issues revealed by a deep investigation of the QoL may lead to modifications in care and/or suggest that some interventions provide little benefit in individuals with DS. The results of the current study suggest that behavioral intervention focused on externalizing symptoms and autistic symptoms could exert a substantial effect on the QoL of children with DS. These findings can be also useful to help families anticipate possible conditions associated with DS and their treatment. Considering research on the QoL of people with DS is still limited (23), the results of the present study could provide crucial evidence for the setting up of proper interventions for children with DS and their families. Specifically, our findings suggest that, beyond medical comorbidities, healthcare professionals should encompass, in their assistance activity, interventions on cognitive functioning, autonomy and challenging behaviors to improve QoL for the children with DS.

## Data availability statement

The raw data supporting the conclusions of this article will be made available by the authors, without undue reservation.

## Ethics statement

The studies involving human participants were reviewed and approved by Bambino Gesù Children's Hospital. Written informed consent to participate in this study was provided by the participants' legal guardian/next of kin.

## Author contributions

Conceptualization and writing—review and editing: EF, FC, and SV. Methodology, formal analysis, and writing—original draft preparation: EF and FC. Investigation and data curation: EF and PG. Supervision and project administration: SV and FC. All authors contributed to the article and approved the submitted version.

## Conflict of interest

The authors declare that the research was conducted in the absence of any commercial or financial relationships that could be construed as a potential conflict of interest.

## Publisher's note

All claims expressed in this article are solely those of the authors and do not necessarily represent those of their affiliated organizations, or those of the publisher, the editors and the reviewers. Any product that may be evaluated in this article, or claim that may be made by its manufacturer, is not guaranteed or endorsed by the publisher.
